# Inject and Sustain Sustainability (iS^2^) in automatic fare collection projects

**DOI:** 10.1186/2193-1801-4-S2-O8

**Published:** 2015-11-27

**Authors:** Hillary Kin-hung Lau, Nicholas Chun-kit Wong

**Affiliations:** School for Higher and Professional Education, Vocational Training Council, Hong Kong; MTR Corporation Limited, Hong Kong

## Background

Sustainable public transport is not an option; it is a necessity [1]. In a media competition run by the Institution of Engineering and Technology (IET), we introduced an award-winning design model of financially secure and sustainable automatic fare collection (AFC) [2]. In the public transport industry, AFC commonly refers to transit providers' automated ticketing systems for collecting fares. We aimed to expand this design model and provide a practical training strategy (incorporating AFC subject knowledge and pedagogic backed training) to answer the challenge of inter-disciplinary capability in knowledge transfer for sustaining sustainability within the project and beyond.

## Methods

The designer evaluates and injects components of sustainability into the design, which is then commissioned. Project management frameworks focus on sustainability insertion and growth. Studies on educational psychology have charted the ideal knowledge transfer pedagogy [3] for sustaining sustainability, but also highlighted the practical challenge of interdisciplinary capability. Case studies on synergy in the Hong Kong vocational training framework and major AFC transport projects validate our practical training strategy.

## Results

This design model does not need to rearrange components in project management - CMMI [4], PRINCE2 [5] - nor does it introduce any unfamiliar components. With such flexibility, projects other than AFC projects can also adopt this model. Identifying tasks that are specific to sustainability and those relating to sustainability in the task breakdown structure is of paramount importance. Integrating tasks with sustainability and with interlocks in task analysis is the next stage. Designing an interdisciplinary pedagogy for complete knowledge transfer will embed sustainability in the system. The synergy of a vocational training provider's collaboration with an industry partner can overcome the challenge of interdisciplinary training. The design model not only sustains the AFC system in the project build but more importantly sustains the injected sustainability beyond the completion of the project.

## Conclusions

Abstraction of these ideas is easier to present than it is to understand. This paper adopts a problem-solving pedagogy to make explicit the design philosophy and model of integrating sustainability. It gives designers a design process guide for injecting sustainability, and implementers of vocational training a model of interdisciplinary collaboration for sustaining sustainability: iS^2^ for tomorrow.

**References**
